# A Simple and Fast Two-Locus Quality Control Test to Detect False Positives Due to Batch Effects in Genome-Wide Association Studies

**DOI:** 10.1002/gepi.20541

**Published:** 2010-11-18

**Authors:** Sang Hong Lee, Dale R Nyholt, Stuart Macgregor, Anjali K Henders, Krina T Zondervan, Grant W Montgomery, Peter M Visscher

**Affiliations:** 1 Queensland Institute of Medical ResearchHerston, Queensland, Australia; 2 Nuffield Department of Obstetrics and Gynaecology, University of Oxford, John Radcliffe HospitalOxford, United Kingdom

**Keywords:** genome-wide association study, batch effects, genotyping errors, linear model-based quality control

## Abstract

The impact of erroneous genotypes having passed standard quality control (QC) can be severe in genome-wide association studies, genotype imputation, and estimation of heritability and prediction of genetic risk based on single nucleotide polymorphisms (SNP). To detect such genotyping errors, a simple two-locus QC method, based on the difference in test statistic of association between single SNPs and pairs of SNPs, was developed and applied. The proposed approach could detect many problematic SNPs with statistical significance even when standard single SNP QC analyses fail to detect them in real data. Depending on the data set used, the number of erroneous SNPs that were not filtered out by standard single SNP QC but detected by the proposed approach varied from a few hundred to thousands. Using simulated data, it was shown that the proposed method was powerful and performed better than other tested existing methods. The power of the proposed approach to detect erroneous genotypes was ∼80% for a 3% error rate per SNP. This novel QC approach is easy to implement and computationally efficient, and can lead to a better quality of genotypes for subsequent genotype-phenotype investigations. *Genet. Epidemiol*. 34:854–862, 2010. © 2010 Wiley-Liss, Inc.

## INTRODUCTION

Genome-wide association studies (GWAS) have been successful in contributing to the dissection of the genetic architecture of complex diseases [WTCCC, 2007]. A crucial step in the analysis of GWAS data is genotype quality control (QC) [Fardo et al., 2009; Mitchell et al., 2003; Pearson and Manolio, 2008]. Among standard QC measures are the exclusion of single nucleotide polymorphisms (SNPs) with low call rates (e.g. <95%) and minor allele frequencies (e.g. <1%), excluding loci due to departures from Hardy-Weinberg equilibrium (HWE) and excluding samples with high missing genotype rates. QC may become more important for more sophisticated analyses including multiple SNP analysis across the whole genome, estimation of heritability from a covariance structure derived from genome-wide SNP data, or prediction of genetic risk based on a set of SNPs that best explains genetic variation [Hoggart et al., 2008; Lee et al., 2008; Manolio et al., 2009; Visscher, 2008; Visscher et al., 2006; Wray et al., 2007].

Erroneous genotypes can be generated from random and/or nonrandom calling errors due to technical problems, DNA quality, DNA sample contamination, or systematic, constant unknown factors such as copy number polymorphisms. Frequently, genotype data from case and control samples are processed separately in different batches due to geographic or time differences when the samples were collected. Therefore, genotype miscalls and errors can be systematically associated with batch effects. Single SNP QC analyses or manual review of cluster plots may identify loci affected by batch effects. SNP QC analyses are routinely performed and implemented in software packages such as PLINK [Purcell et al., 2007]. Routine whole-genome QC analyses using multiple SNPs are less common.

In this report, a simple two-locus linear model analysis is used to detect SNP genotyping errors that are likely due to batch effects. The proposed approach can detect problematic SNPs with statistical significance even when standard single SNP QC analyses fail to detect them. With this novel approach, which is easy to implement and computationally fast, genotype quality can be improved.

## RATIONALE

Standard single SNP QC analyses were performed on genome-wide SNP data from women recruited in Australia for a study of endometriosis [Treloar et al., 2002, 2005] and Australian controls genotyped in the same laboratory in a separate project [Ferreira et al., 2010; Medland et al., 2010]. A quantile-quantile (QQ) plot from preliminary analysis of single SNP association provided no evidence for systematic deviation from the null distribution of no association. We then fitted two-locus models to see if they would provide stronger evidence for association than the single-locus models. Unexpectedly, we found highly significant signals for pairs of SNPs in linkage disequilibrium, well beyond what might be expected for true association signals. We investigated the reason for this substantial difference between the single and multiple SNP test and concluded (see below) that the unusual difference between the tests was because of genotyping errors and batch effects.

**Fig. 1:**
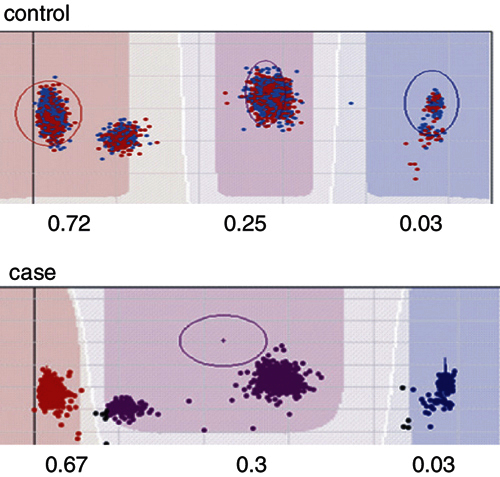
Genotype miscalls in an opposite direction in cases and controls. Some of the second cluster were called as homozygous in the controls, whereas they were called as heterozygous in cases. The proportion of the samples in each group is given below each cluster.

As an example, Figure 1 shows a genotype cluster plot for a SNP (called SNP1) that passed standard QC before inspection of cluster plots, but gave an unusually large test statistic for a two-locus test of association. The SNP has four genotype clusters in both cases and controls, but in the control group the second cluster was called as homozygous, whereas in the case group it was called as a heterozygote. In Table I, the genotype frequencies of SNP1 are shown. The total number of samples was 4,010. Using a linear model, the single SNP test for association gave a log-likelihood ratio (LR) of 20.05 (*P*-value = 7.5e−06). When considering an adjacent SNP (SNP2), the genotype frequencies in the controls were slightly different from those of SNP1 and the single-locus association test gave a LR of 1.32 (*P*-value = 0.25) for SNP2 (Table I). For an additive model, we expect that the association test for the combined genotypes of SNP1 and SNP2 (diplotypes) would give a similar value to the sum of the LR for each single SNP test, i.e. LR(SNP1,SNP2)≍LR(SNP1)+LR(SNP2). However, the association test for combined genotypes of SNP1 and SNP2 gave a LR of 124.06 (*P*-value = 1.15e−27) (Table I). One combination of the genotypes for SNP1 and SNP2, (T,T) (G,T), was 24 times more frequent in the controls than in the cases (Table I). Given the cluster plots in Figure 1, it is likely that the genotype (T,T) for SNP1 associated with the genotype (G,T) for SNP2 was generated due to genotype call errors. This hypothesis is supported by a haplotype analysis. The frequency of the haplotype TT was much higher in the controls than in the cases, resulting in high significance for a haplotype association test (*P*-value = 8.36e−29) (Table I). However, a combination of the haplotype TT and TT was not observed at all in this sample, which strongly supported that the haplotype TT was artificially generated.

**TABLE I:** Genotype, diplotype and haplotype frequency for SNP1 and SNP2 in cases and controls

	Cases	Controls
*Genotypes for SNP1*		
T,T	0.665	0.731
T,C	0.306	0.244
C,C	0.029	0.023
*P*-value^a^	7.5e−06	
*Genotypes for SNP2*		
G,G	0.665	0.676
G,T	0.306	0.301
T,T	0.030	0.023
*P*-value^a^	0.25	
*Diplotypes for SNP1 and SNP2*		
(T,T) (G,G)	0.663	0.676
(T,T) (G,T)	0.002	0.057
(TT) (T,T)	0.000	0.000
(T,C) (G,G)	0.002	0.000
(T,C) (G,T)	0.305	0.244
(T,C) (T,T)	0.000	0.000
(C,C) (G,G)	0.000	0.000
(C,C) (G,T)	0.000	0.000
(C,C) (T,T)	0.029	0.023
*P*-value^a^	1.15e−27	
*Haplotypes for SNP1 and SNP2*		
TG	0.817	0.827
CT	0.182	0.145
TT	0.001	0.028
*P*-value^a^	8.36e−29	

a *P*-value for significance of the frequency difference between cases and controls.

Since both SNPs passed standard QC, we aimed to develop a novel QC test to detect such genotyping errors and thereby reduce false positives due to batch effects.

## METHODS

Linear models were used to detect potential false positives due to genotyping errors. A null model without any SNP effect is,


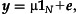
(1)

where ***y*** is a vector of *N* phenotypic observations, µ is the overall mean, **1**_***N***_ is a vector of *N* ones and ***e*** is a vector of residuals which are assumed to be normally distributed with mean zero and variance 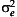. In case-control studies, *y* has values of e.g. 0 and 1, whereas for quantitative traits the *y* values are continuous. When testing the effect of the ith SNP, the following additive model can be used,


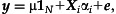
(2)

where ***X***_***i***_ is a vector of coefficients 0, 1, or 2 representing indicator variables of the genotypes of the *i*th SNP, and α_*i*_ is the effect of the *i*th SNP. When considering joint effects from the *i*th SNP and *j*th SNP, the model can be extended as,


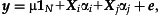
(3)

with ***X***_***j***_ a vector of the genotype coefficients for the *j*th SNP, and α_*j*_ the effect of the *j*th SNP.

The natural logarithm of the likelihood given the model parameters for (2) is, assuming that the residuals are normally distributed,


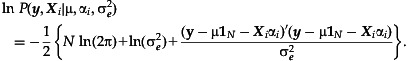
(4)

The likelihood function for (1) and (3) can be accordingly derived (not shown). The logarithm of the likelihood ratio (LR) for the model fitting the *i*th SNP effect compared to the null model, is


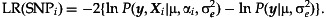
(5)

The LR for the model fitting joint effects from the *i*th SNP and *j*th SNP, compared to the null model, is defined as,


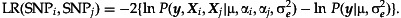
(6)

From (5) and (6), an adjusted likelihood ratio (LR*) can be constructed for SNP_i_ to test the difference in fit between the two-locus and single-locus models,


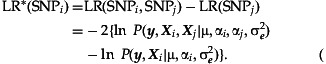
(7)

Under the assumption of normality, the above LR test statistics are simple functions of the residual sums of squares from least squares linear regression. The value for LR*(SNP_*i*_) is χ^2^-distributed with one degree of freedom when the SNPs are uncorrelated and in the absence of genotyping artifacts.

To implement the LR*(SNP_*i*_) test genome-wide, a sliding window approach was taken in which pairs of adjacent SNPs were used in (7).

### SIMULATION STUDY

Coalescence simulations were carried out using the program “ms” [Hudson, 2002] to generate SNPs under a Fisher-Wright equilibrium model. Parameters for the simulation of 2-Mb regions of the genome were chosen consistent with an effective population size *N*_*e*_ = 10,000, a mutation rate τ = 10e−08/site/generation, and a recombination rate *r* = 10e−08/base pair/generation. Therefore, the parameters used for the program were θ = 4 *N*_e_*τ*2,000,000 = 800, and ρ = 4 *N*_e_*r*2,000,000 = 800. For each replicate, 109 polymorphic sites were selected such that the average marker interval was ∼0.01 Mb, and the minor allele frequency (MAF) for each SNP was more than 0.05. The number of samples was 10,000, and 1,000 cases and 1,000 controls were randomly selected for the analysis.

For simulating higher levels of LD between SNPs, the effective size, mutation rate or/and recombination rate were reduced. We used θ = ρ = 560, 320, or 80 for alternative levels of LD.

#### Genotyping error model

Genotyping errors were introduced to every 10th SNP. Therefore, 10 SNPs of 109 SNPs had erroneous calls in each replicate. For the analysis of statistical power to detect genotyping errors, the 10 SNPs with errors and adjacent SNPs were used. The 10 SNPs with errors were in approximate linkage equilibrium.

Six kinds of error models were considered (Table II). For error models 1–4, 10% of either the cases or controls were randomly selected and their genotypes altered. In the first error model (error 1), if a selected individual was heterozygous, the genotype was switched to homozygous for the major allele. In the second error model (error 2), if a selected individual was homozygous for the major allele, the genotype was switched to heterozygous. In the third error model (error 3), if a selected individual was heterozygous, the genotype was switched to homozygous for the minor allele. In the fourth error model (error 4), if a selected individual was homozygous for the minor allele, the genotype was switched to heterozygous. In the fifth and sixth error model, genotyping errors occur in both case and control groups with different directions mimicking the observed example (Fig. 1), and 5% of the individuals were randomly selected in each group and their genotypes manipulated. In the fifth error model (error 5), heterozygous genotypes for selected individuals were switched to homozygous for the major allele in one group, whereas homozygous genotypes for the major allele for selected individuals were switched to heterozygous in the other group. In the sixth error model (error 6), heterozygous genotypes for selected individuals were switched to homozygous for the minor allele in one group, whereas homozygous genotypes for the minor allele for selected individuals were switched to heterozygous in the other group.

**TABLE II:** Error model names and details

Model name	Details
Error 1	Heterozygous miscalled as homozygous for major allele in random 10% of either cases or controls
Error 2	Homozygous for major allele miscalled as heterozygous in random 10% of either cases or controls
Error 3	Heterozygous miscalled as homozygous for minor allele in random 10% of either cases or controls
Error 4	Homozygous for minor allele miscalled as heterozygous in random 10% of either cases or controls
Error 5	Heterozygous miscalled as homozygous for major allele in random 5% of cases, and homozygous for major allele miscalled as heterozygous in random 5% of controls
Error 6	Heterozygous miscalled as homozygous for minor allele in random 5% of cases, and homozygous for minor allele miscalled as heterozygous in random 5% of controls

#### Power calculation using simulated data

For comparisons, four existing methods were used to calculate the power using simulated data. First, the single SNP association test (ssa) was used. Second, the linear model-based QC (lmq), the proposed method in this study, was used. Third, a haplotype association test (hap) was used where haplotypes for pairs of adjacent SNPs were estimated using an expectation-maximization algorithm, and an association test for the haplotypes was carried out [Purcell et al., 2006, 2007]. Finally, a LD-contrast (ldc) method was used to test the significance of the difference for *r*^2^(LD measure) of a pair of adjacent SNPs between cases and controls [Zaykin et al., 2006]. The computer programs PLINK [Purcell et al., 2007] and LD-contrast [Zaykin et al. 2006] were used for the hap and ldc analyses, respectively.

The type-I error was calculated from 10,000 tests with simulations of no genotyping errors. Empirical power was calculated from 1,000 tests with simulations for the genotyping error models, using the type-I error thresholds generated under the simulations without genotyping errors.

### REAL DATA

The lmq test was further applied to real data. We analyzed genome-wide SNP data from a population-based case-control study for endometriosis [Painter et al.,2010, submitted; Treloar et al., 2002]. Our main interest was to detect possible false positives due to batch effects. For comparison, two independent control samples from Welcome Trust Case Control Consortium (WTCCC) were used for the analysis [WTCCC, 2007]. One of the two control groups was treated as a case group and the other was treated as a control group in the analysis.

For both data sets, the same standard QC analysis was performed. SNPs with MAFs<0.01, missing rates >0.05, and *P*-values for HWE <0.0001, and individuals with missing rates >0.01 were filtered out. Sex chromosomes were excluded from the analysis. After the QC process, 4,083 individuals (2,247 cases and 1,836 controls) with 496,733 SNPs were used for the analysis of the endometriosis data, and 2,439 individuals (1,186 cases and 1,253 controls) with 385,054 SNPs were used for the analysis of the WTCCC data.

Four different threshold values were used to filter out SNPs with high missing rates, i.e. 0.05, 0.02, 0.01, and 0.001. For each threshold value, the inflation factor of the test statistics, the number of inflated data points, and QQ plots for ssa and lmq were reported as indicators of data quality.

## RESULTS

### SIMULATION STUDY

#### Type-I error and power using simulated data

When there was no genotyping error, the Type-I error rate was maintained at acceptable levels for all methods using simulated data with both situations of low LD (simulation parameters θ = ρ = 800) and high LD (θ = ρ = 80) (Tables III and IV). For the situation of high LD, the values for the type-I error rate were slightly low for lmq and ldc. This is to be expected because when adjacent SNPs are in high LD, the single and pairwise SNP tests for association become more similar, so that their difference does not follow a χ^2^ with one degree of freedom. In the extreme case of pairs of SNPs being in perfect LD, the single and pairwise tests are identical and the type-I error rate assuming a χ^2^ would be zero.

**TABLE III:** Type I error and empirical power when using simulation parameters θ = ρ = 800

	Error models						
	
Methods	No error^a^	Error 1	Error 2	Error 3	Error 4	Error 5	Error 6
ssa	0.054	0.255	0.670	0.226	0.063	0.529	0.115
lmq	0.050	0.335	0.736	0.309	0.082	0.617	0.176
hap	0.052	0.245	0.647	0.220	0.048	0.413	0.089
ldc	0.053	0.160	0.246	0.144	0.091	0.110	0.097

The parameters, θ = ρ = 800, generate low LD.

a Type-I error rate.

**TABLE IV:** Type-I error and empirical power when using simulation parameters θ = ρ = 80

	Error models						
	
Methods	No error^a^	Error 1	Error 2	Error 3	Error 4	Error 5	Error 6
ssa	0.049	0.241	0.678	0.197	0.049	0.518	0.136
lmq	0.039	0.432	0.787	0.373	0.162	0.654	0.315
hap	0.044	0.274	0.676	0.235	0.047	0.416	0.096
ldc	0.041	0.278	0.386	0.262	0.160	0.160	0.188

The parameters, θ = ρ = 80, generate high LD.

a Type-I error rate.

Generally, the most powerful approach was lmq for which the power ranged from 0.08 to 0.74 for low LD (Table III) and from 0.16 to 0.79 for high LD (Table IV). The powers for ssa and hap methods were similar to each other when using error models 1 to 4. However, when using error models 5 and 6, where genotyping errors occurred in both cases and controls, ssa was more powerful than hap. The power for ldc was relatively low.

#### Genotyping error rate and power

**Fig. 2:**
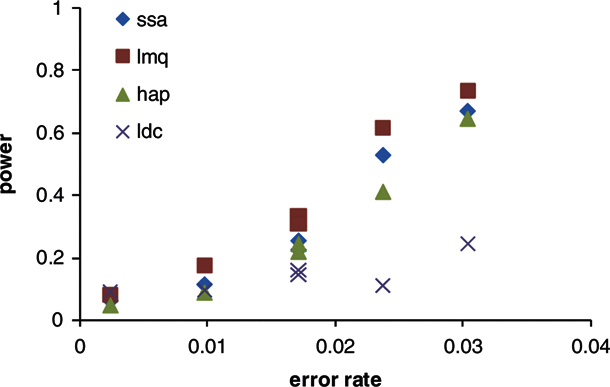
Increase in the power with higher genotyping error rates. Error model 1: 1.7%, error model 2: 3%, error model 3: 1.7%, error model 4: 0.2%, error model 5: 2.3%, error model 6: 1%.

For the simulated data, the average MAF of the selected SNPs was ∼0.2. The genotyping error rate per SNP was estimated for each error model. The error rate was 1.7, 3, 1.7, 0.2, 2.3, and 1% for the error models 1, 2, 3, 4, 5, and 6, respectively. Figure 2 shows that the power generally increases when the genotyping error rate increases as expected. For lmq, the power increased up to 75% when the genotyping error rate was about 3% (Fig. 2).

#### Levels of LD and power

**Fig. 3:**
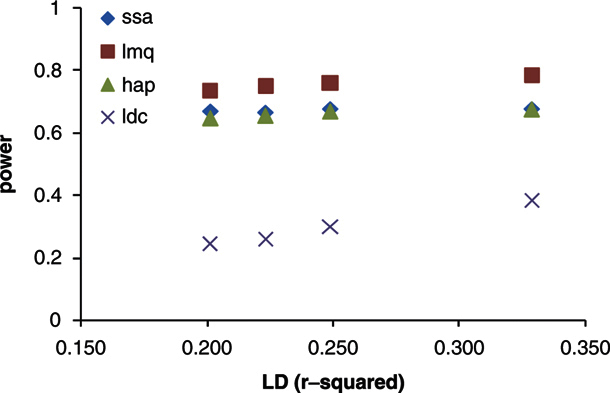
Slight increase in the power with higher levels of LD using error model 2.

When using simulation parameters θ = ρ = 800, 560, 320, and 80 with the error model 2 (homozygous for major allele switched to heterozygous), the estimated *r*^2^ for adjacent SNPs was 0.20, 0.22, 0.25, and 0.33, respectively (Fig. 3). The power slightly increased when the level of LD increased, e.g. for lmq, the power increased from 0.74 to 0.79 (Fig. 3), but this increase was modest.

### REAL DATA

For the endometriosis data, the overall quality of the genotypes was high. As expected, the proportion of excluded SNPs gradually increased when the threshold value for missing rates per SNP decreased (Table V). However, even with a stringent threshold value, a relatively small number of SNPs were excluded, i.e. 0.2, 0.6, and 8.6% of 496,733 SNPs for threshold values of 0.02, 0.01, and 0.001 (Table V). For the WTCCC data, the proportion of excluded SNPs was relatively large, i.e. 5, 13, and 48% of 385,054 SNPs for threshold values of 0.02, 0.01, and 0.001 (Table V).

**TABLE V:** The number of SNPs, test statistic inflation factor λ, and the number of data points deviating from expectation for each threshold value for the endometriosis data and the WTCCC data

		Single SNP analysis		lmq analysis	
			
Missing rate threshold	No. SNP	λ	No. data (%)	λ	No. data (%)
*Endometriosis data*					
0.05	496,733	1.023	28 (0.01)	1.025	228 (0.05)
0.02	495,911	1.023	28 (0.01)	1.025	193 (0.04)
0.01	493,289	1.022	23 (0)	1.025	52 (0.01)
0.001	454,193	1.023	25 (0.01)	1.023	1 (0)
*WTCCC data*					
0.05	385,054	1.032	5,019 (1.3)	1.102	29,275 (7.6)
0.02	366,440	1.018	68 (0.02)	1.071	11,707 (3.2)
0.01	336,850	1.012	0 (0)	1.049	2,375 (0.7)
0.001	201,620	1.012	0 (0)	1.017	8 (0)

**Fig. 4:**
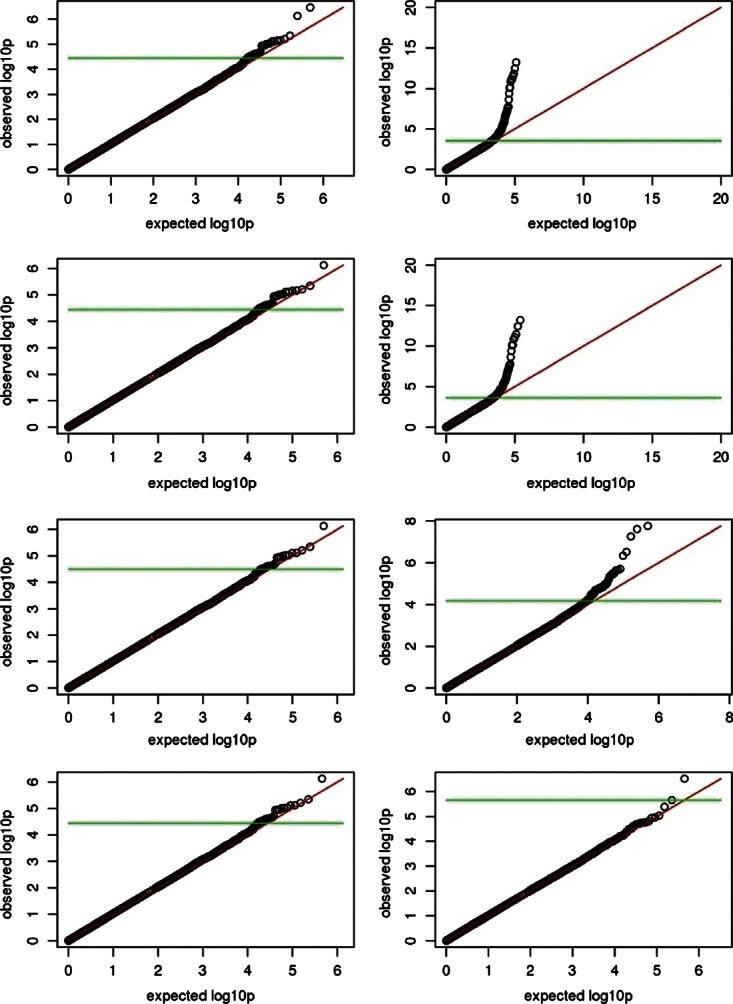
QQ plots of the negative logarithm of *P*-value when using endometriosis data. The left column is for ssa and the right column is for lmq in a vertical order of the applied threshold 0.05, 0.02, 0.01, and 0.001. The horizontal line is determined such that the difference between the negative logarithms of the expected and observed *P*-value above the line is greater than 0.2. QQ, quantile-quantile.

The number of data points is counted for the values exceeding the horizontal line in each graph in Figures4 (endometriosis) and 5 (WTCCC). The number of data proportional to the number of SNPs is shown as percentage in bracket.

Figure 4 shows QQ plots for the ssa tests (left column) and for the pairwise lmq test (right column) in a vertical order of the applied threshold 0.05, 0.02, 0.01, and 0.001 when using the endometriosis data. With the threshold values of 0.05, 0.02, or 0.01, the patterns of the plots for ssa differed from those for lmq. For ssa, the plots showed a very good agreement between the expected and observed values and the inflation factor (λ) was close to one, and few data points deviated from expectation (data points above the horizontal line) (Table V). However, the plots for lmq showed that observed values deviated from expectation for a relatively large number of points (Fig. 4 and Table V). Deviation from expectation is likely to occur because of genotyping errors due to batch effects. More stringent threshold values for missing rates per SNP reduced the number of data points that deviated from expectation. There were few data points that deviated from expectation with the threshold of 0.001 (Fig. 4 and Table V).

**Fig. 5:**
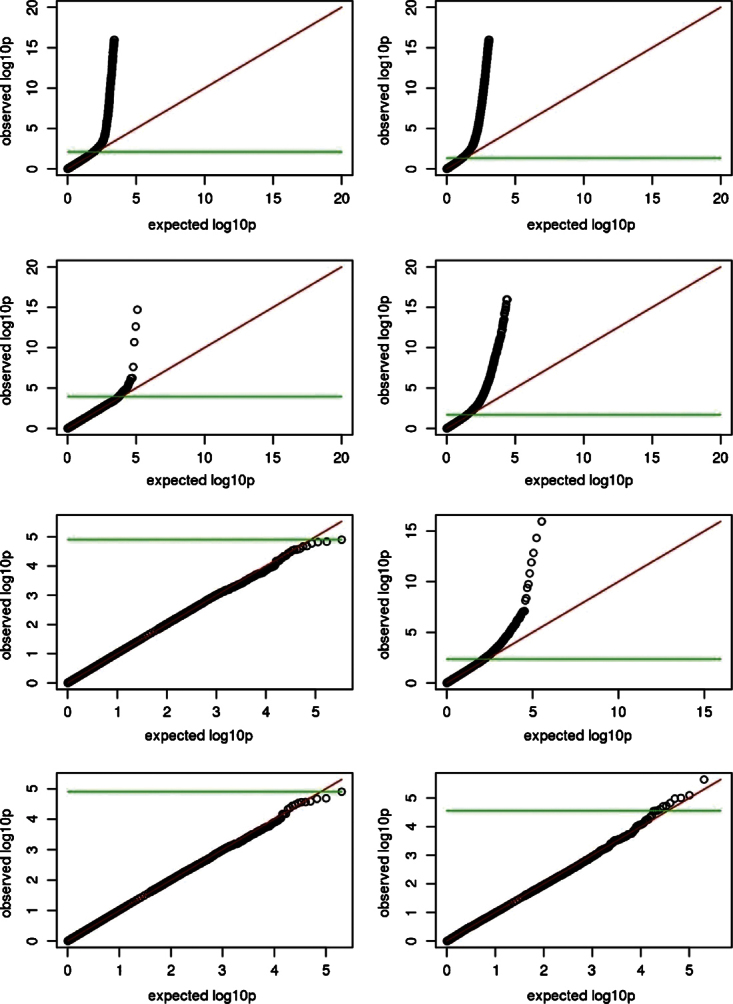
QQ plots of the negative logarithm of *P*-value from when using WTCCC data. The left column is for ssa and the right column is for lmq in a vertical order of the applied threshold 0.05, 0.02, 0.01, and 0.001. The horizontal line is determined such that the difference between the negative logarithms of the expected and observed *P*-value above the line is greater than 0.2. QQ, quantile-quantile.

When using the WTCCC data, the number of data points that deviated from expectation was large unless SNPs having missing rates of more than 0.01 were filtered out (Fig. 5). The difference between the results from ssa and lmq was remarkable. With the threshold value of 0.05, the number of data points deviating from expectation was 5,019 (1.3% of the corresponding number of SNPs) for ssa and 29,275 (7.6%) for lmq (Table V). With lower threshold values, the number of SNPs that deviated from expectation was reduced. With the threshold value of 0.01, there was a good agreement between the expected and observed values for ssa. However, there was still significant deviation for lmq. For example, the number of data points that deviated from the expectation was 2,375 (0.7%). This indicated that there were still genotyping errors due to batch effects although the proportion was low. Few data points deviated from expectation when using a stringent threshold value of 0.001 (Fig. 5 and Table V). The inflation factor (λ) was close to 1.0 for lower threshold values for both ssa and lmq, although the pattern was more obvious for lmq.

## DISCUSSION

We have developed and applied a novel lmq method, and showed that it could detect many of the problematic SNPs that already passed standard single SNP QC filters. Using simulated data, we showed that the lmq test was reasonably powerful and performed well in comparison with other methods.

In GWAS, the impact of erroneous genotypes can be severe on false-positive SNP-phenotype associations, estimation of heritability, and prediction of genetic risk. Erroneous SNPs with high significance may be accepted as genuine if they have already passed a standard QC process although manual inspection of cluster plots will detect some problematic SNPs. Even if the significance of SNPs with erroneous genotype calls is low enough to be ignored, accumulated information from such SNPs can cause high significance for haplotype-based association tests, overestimation of relationships between individuals within each case and control group, and inaccurate and imprecise prediction of genetic risk [Becker et al., 2006; Liu et al., 2006; Marquard et al., 2009]. Furthermore, meta-analysis from large multi-centre studies relies heavily on genotype imputation to create a set of genotypes (or dosage scores) that can be compared across studies. Genotyping errors such as detected by our method could, if undetected, lead to an increased rate of false positives and false negatives.

We used a simple linear model assuming that the residuals were normally distributed. In a more formal way, a non-linear model such as a logistic regression could be used to test the difference in fit between the two-locus and single-locus models. However, the obtained LR values between a linear and logistic model were very similar (result not shown). We prefer the simple linear model because of its computational efficiency.

Although we did not simulate a disease-association model, we applied the method to the endometriosis data. For those data, we observed that the SNPs detected by the two-locus QC were obviously problematic in their cluster plots and GenTrain scores. Conversely, we observed that the lmq test statistics for SNPs having high significance for disease-association with clear cluster plots and high GenTrain scores were not inflated compared to the ssa test statistic (the best 10 SNPs were checked). This indicated that at least for these data the two-locus QC test performed properly well, in that it detected false positives (as evidenced by a closer inspection of the SNP quality scores) yet did not flag SNPs that appear to be associated with disease.

The two-locus QC detects hidden (rare) haplotypes across two loci that are associated with disease status. It is true that such haplotypes could be generated due to genuine causal variants (e.g., recent mutations). To minimize false positives or discarding truly associated SNPs, we used a composite strategy to control data quality, i.e. removing problematic SNPs based on SNP missing rates and assessing data quality by the lmq statistics (Table V and Figs. 4 and 5).

In order to remove problematic SNPs, it should be safe and reasonable to rely on the information of SNP missing rates as low-quality genotypes often result in high missing rates (low call rates) [Fu et al., 2009]. We used four thresholds for missing rates per SNP and indicated that the statistical signals for the presence of erroneous genotypes clearly reduced with more stringent thresholds. A high restriction allowing only minimum missing rates may exclude SNPs that do not suffer from genotyping errors. However, the loss of information is not large. For example, for the endometriosis data, the restriction of missing rates per SNP less than 0.01 excluded only ∼1% of 496,733 SNPs that passed a preliminary standard QC.

We checked the performance of the proposed approach compared to GenTrain and cluster separation scores that can be obtained from the Illumina Beadstation software. We investigated the most problematic 11 pairs of SNPs according to their LR from the lmq test using the endometriosis data. Usually, only one SNP of each pair appeared to cause the inflated test statistic. Details of these 11 SNPs are listed in Table VII. In general, the GenTrain scores were reasonably high for all the SNPs (above 0.7). This was expected because the listed SNPs already passed standard QC. The cluster separation scores were low for most of the problematic SNPs, but high for rs4621162 and rs10810402. Interestingly, the missing rates were relatively high (>0.01) for all the flagged SNPs.

**TABLE VI:** The number of SNPs significant at *P*-value thresholds of 0.05 and 0.01 from the lmq or the PLINK test-missing function for the endometriosis data

lmq	test-missing	*P*-value<0.05	*P*-value<0.01
Significant			
Yes	Yes	1,091	202
Yes	No	25,010	5,221
No	Yes	15,878	9,648
No	No	454,753	481,661

The total number of SNP is 496,733, and the number of SNP with a missing rate >0 for both cases and controls is 258,628.

**TABLE VII:** GenTrain scores and cluster separation scores for the most problematic 11 SNPs identified by the lmq analysis

		Likelihood ratio		GenTrain score		Separation score		
					
chr	SNP	ssa	lmq	Case	Control	Case	Control	Missingness
1	rs9970392	8.140	49.900	0.775	0.813	0.241	0.524	0.017
2	rs4621162	6.700	43.340	0.766	0.788	0.958	0.861	0.041
4	rs3924900	0.230	89.748	0.808	0.832	0.337	0.540	0.020
4	rs7689397	4.784	42.214	0.851	0.852	1.000	0.526	0.021
6	rs204990	0.215	43.923	0.838	0.887	0.437	0.765	0.016
8	rs4738899	1.487	52.875	0.803	0.848	0.405	0.589	0.018
9	rs10810402	20.180	78.988	0.844	0.906	1.000	0.950	0.016
9	rs7862315	6.116	48.435	0.765	0.836	0.372	0.669	0.015
11	rs10834007	0.387	35.344	0.803	0.882	0.320	0.801	0.020
13	rs9596897	0.423	42.379	0.810	0.860	0.384	0.701	0.014
13	rs1330047	0.000	39.074	0.806	0.824	0.359	0.513	0.014

Are there other simple tests readily available that would detect the same problematic SNPs? The versatile program PLINK has a number of functions that would be implemented for QC. For example, the function loop-assoc can be used to test for an association between gene frequency and plate. The PLINK function test-missing tests the difference of missing rate per SNP between cases and controls. We applied this function to the endometriosis data (Table VI). We observed that a large proportion of problematic SNPs detected by our two-locus test showed no significance for the difference of missing rate. It is noted that the PLINK-test-missing function may not be able to pick up differential haplotypic missingness such as error models 5 and 6. On the other hand, a large proportion of SNPs not identified by our two-locus test gave significance for the difference of the missing rate between cases and controls. There were not many common SNPs that were picked up by both the proposed method and PLINK test-missing function (Table VI). Therefore, at least for the endometriosis data, the test for differential missingness across cases and controls identifies a different subset of potential problematic SNPs than our proposed method.

We used a sliding window approach fitting pairs of SNPs starting from one end of the chromosome. We checked how stable the results were if it started from the other end using the endometriosis data. When carrying out the lmq analysis with a sliding window from pter (short arm telomeric end) to qter (long arm telomeric end) or from qter to pter, the test statistic inflation factor λ and the number of data points deviating from expectation for each threshold value were very similar (Table VIII). This indicates that the method appears robust to sliding window orientation.

**TABLE VIII:** Test statistic inflation factor λ and the number of data points deviating from expectation for each threshold value for the endometriosis data when carrying out the lmq analysis with a sliding window from pter to qter or from qter to pter

	pter to qter		qter to pter	
		
Missing rate threshold	λ	No. data (%)	λ	No. data (%)
0.05	1.025	228 (0.05)	1.028	228 (0.05)
0.02	1.025	193 (0.04)	1.027	193 (0.04)
0.01	1.025	52 (0.01)	1.027	52 (0.01)
0.001	1.023	1 (0)	1.025	0 (0)

In conclusion, we propose a simple and fast two-locus test to detect SNPs among those that passed single-SNP QC that are likely to suffer from batch effects. The proposed test harvests haplotypic information to provide a sensitive approach to identify differential technical artefacts across genotyping projects/batches that is not detected using current standard QC of GWA data. In combination with standard single-SNP QC analyses, our test may contribute to a better quality of the genotypes used for subsequent genotype-phenotype investigations.
